# Aging induces T cells with distinct transcriptomic profiles and functions in brain-associated tissues

**DOI:** 10.3389/fimmu.2025.1619196

**Published:** 2025-06-04

**Authors:** Youwen Si, Yuanyue Zhang, Qi Yang

**Affiliations:** ^1^ Child Health Institute of New Jersey, Rutgers Robert Wood Johnson Medical School, New Brunswick, NJ, United States; ^2^ Rutgers Institute for Translational Medicine and Science, Rutgers Robert Wood Johnson Medical School, New Brunswick, NJ, United States; ^3^ Department of Pediatrics, Rutgers Robert Wood Johnson Medical School, New Brunswick, NJ, United States

**Keywords:** T cell, CD153, aging, cognitive function, CD4, CD8

## Abstract

**Background:**

Aging is known to induce the emergence of distinct lymphocyte populations with unique molecular and functional characteristics. However, the impact of aging on the transcriptomes and functional activities of CD4 and CD8 T cells in non-lymphoid tissue remains poorly understood. Investigating aging-induced transcriptomic changes in tissue-infiltrating immune cells may provide insights into tissue homeostasis and malignancy in the aging context.

**Methods:**

Single-cell RNA sequencing (scRNA-seq) was performed to compare the cell subsets and transcriptomes of CD4^+^ and CD8^+^ T cells in brain-associated tissue, including the meninges and choroid plexus of young and aged mice. Flow cytometry was used to analyze aging-associated CD4^+^ T cells in the hippocampus. Depletion antibodies were employed to investigate the functional role of aging-associated T cells.

**Results:**

Aging induces a shift in the transcriptomes of CD4^+^ and CD8^+^ T cells in the meninges and choroid plexus toward an effector memory phenotype. In aged mice, T helper 2 (Th2) cells, regulatory T cells (Tregs), and distinct subsets of CD153-expressing CD4^+^ T cells accumulate in these brain-associated regions. Notably, CD153-expressing CD4^+^ T cells also infiltrate the hippocampus. Depletion of CD153^+^ cells using anti-CD153 antibodies leads to impaired cognitive function, suggesting a potential protective role for these cells in the aging brain.

**Conclusions:**

Aging alters the transcriptome of brain-associated CD4^+^ and CD8^+^ T cells. In particular, distinct CD153-expressing CD4^+^ T cells accumulate in the meninges and choroid plexus, and also infiltrate the hippocampus during aging. These cells may play a protective role in maintaining brain homeostasis.

## Background

Aging is a multifaceted biological process that profoundly impacts the mammalian immune system. Aging leads to both a decline of immune defense to pathogens and malignancies, and elevated tissue inflammation. Aging leads to reduced output of naïve T cell and B cell generation and contracted TCR/BCR repertoires (1). However, the profound effects of aging on lymphocyte development and function are far more complex than pan-inhibition. Notably, aging is associated with the emergence of distinct subsets of T cells, B cells, and innate lymphocytes, whose immunological and functional roles remain incompletely defined (2–7). Some of these aging-associated changes might contribute to sustained tissue inflammation. Nevertheless, regulatory T cells (Tregs) and other immunomodulatory or tissue-reparative immune cell populations also increase with age, potentially representing compensatory mechanisms aimed at preserving physiological homeostasis (8, 9).

Growing evidence from recent studies has highlighted a close interplay between the immune system and the brain (10–15). Immune cells are particularly enriched in the brain barrier regions, including the blood brain barriers (BBB), choroid plexus and meninges (17, 18). In addition, non-microglial immune cells, such as CD8^+^ T lymphocytes, may infiltrate the brain parenchyma in pathological conditions and contribute to tissue inflammation and neurodegeneration (16–20). The specific effects of aging on the transcriptomes, infiltration and functional states of brain-associated lymphocytes remain incompletely understood. While single-cell RNA-seq has been performed with whole meningeal cells including T cells in young and aged mice (21), the analysis for CD4 and CD8 T cells subsets was too broad and lacked in-depth investigation of distinct aging-associated T cell subsets. In addition, the impact of aging on T cell responses in other brain-associated compartments, such as the choroid plexus and brain parenchyma, has yet to be elucidated.

In this study, we examined the single-cell transcriptomes of CD4 and CD8 T cells in the meninges and choroid plexus (22) of young and aged mice. Our data suggest CD8 and CD4 T cells in the brain barrier regions of aged mice exhibit effector memory phenotype. In particular, Th2 cells, Tregs and distinct subsets of CD153+ T cells accumulated in both meninges and CP with aging. Flow cytometry analyses showed that CD153+ T cells also infiltrated into the brain parenchyma in aged mice. Depletion of CD153+ cells using depleting antibodies led to worse cognitive function. Together, our data profiled the transcriptomic changes in brain-associated T cells, identified unique aging-associated CD153+ cells in the brain border and parenchyma regions, and suggest a potentially protective role for these cells.

## Methods

### Animals

Young (10-week) and aged (18 month) female C57BL/6 mice were obtained from the National Institute of Aging via Charles River. Sex-matched mice were randomly assigned to experimental and treatment groups. Experimenters were not blinded to the experimental groups. The animal study was approved by Institutional Animal Care and Use Committee at Rutgers University. The study was conducted in accordance with the local legislation and institutional requirements.

### Antibody treatment and behavior tests

Anti-CD153 (Clone RM153) and isotype controls were purchased from Thermo. Mice were treated with intraperitoneal (i.p.) injections of 0.5 mg anti-CD153 antibodies or isotype controls once every 3 days for 8 weeks. Mice were 18-month old at the time of antibody treatment, and 20 months old at the end of the behavior tests. Specific time frame for antibody treatment and behavior tests were described in **Supplementary Figure 1**. Behavioral testing was conducted as previously described (11, 23–25). All tests were recorded and automatically scored using ANY-maze software (Stoelting Co.). Data were recorded and analyzed by the ANY-maze software (Stoelting Co.). For Open Field test, each mouse was placed in the southeast corner of a 50 × 50 cm white square arena and allowed to explore freely for 10 minutes. Water Maze Testing took place in a round pool with a 125 cm diameter, filled with water made opaque using non-toxic white paint and kept at a temperature of 21–22°C. Visual markers were positioned on all four walls surrounding the pool, which was divided conceptually into four quadrants. During the training phase (Days 1–4), an invisible platform was hidden 1 cm beneath the surface in the southeastern quadrant. Mice were released from alternating starting points located across from the platform’s quadrant. A trial concluded once the mouse located the platform and stayed on it for 10 seconds. If unsuccessful within 60 seconds, the mouse was guided to the platform and allowed to rest there for 10 seconds. Each mouse completed four 1-minute trials per day for four consecutive days. On the fifth day, the platform was removed for the probe test, and mice were allowed to swim freely for 60 seconds. Two independent behavior tests were performed.

Novel object tests were performed in separate behavior tests. For Novel Object Test, mice were habituated to the open field for 10 mins on day 1 as described above. On day 2, two identical objects were placed in the arena and mice were allowed to explore for 10 mins. 24 hours later, one of the objects was replaced with a novel object, and mice were allowed to explore for 10 mins. Exploration was defined as orientation toward the object within a 2 cm distance, as well as direct interactions such as climbing, sniffing, or pushing. Novel object recognition was assessed by calculating the percentage of time spent exploring the novel object relative to the total exploration time of both objects. Two independent behavior tests were performed.

### Flow cytometric analysis and fluorescence activated cell sorting

Flow cytometric analysis and FACS sorting were performed as we previously described (23). Briefly, tissues including the outer dorsal meninges, inner calvaria surface, choroid plexus (22), and hippocampus were dissected on ice. Samples were digested for 20 minutes at 37°C in HBSS containing 0.2 mg/mL Liberase TM and 0.1 mg/mL DNase I. For analysis of meningeal and CP cells, the digested tissue was passed through a 70 µm cell strainer to obtain a single-cell suspension. For hippocampal T cell isolation, enzymatic digestion was followed by Percoll gradient centrifugation to remove debris. Cells were stained with surface antibodies for 30 minutes on ice. The following antibodies were used: anti-CD3ϵ (145-2C11), anti-CD19 (ID3), anti-CD4 (GK1.5), anti-CD11b (M1/70), anti-CD45 (104), anti-CD8 (YTS156.7.7), and anti-CD153 (RM153). Dead cells were excluded using DAPI (Thermo Fisher). Flow cytometry was conducted on a 4-laser LSRII (BD Biosciences) or a 4-laser Cytek Aurora system. Cell sorting was performed using a Cytek Aurora CS cell sorter.

For intravascular labeling of CD45, mice were injected intravenously with 3 µg of anti-CD45.2 PE and euthanized 3 minutes later, as previously described (26). Flow cytometry analysis was conducted as described above.

### Single cell RNA-seq

For single cell RNA sequencing, CD4 and CD8 T cells were sorted from the meninges and choroid plexus of young and aged mice by flow cytometry analysis. scRNA-seq was performed as we previously described (11, 25, 27). Libraries were prepared using the Chromium 5’ Single Cell Gene Expression Kit (10x Genomics) following the manufacturer’s protocol, and sequencing was carried out on an Illumina NextSeq 500 platform. Preliminary data processing was performed using Cell Ranger. Cells with fewer than 200 but more than 7000 genes, or more than 5% of mitochondrial genes, were filtered out. Doublets were removed using the DoubletFinder R package. Data were log-normalized and scaled using Seurat (28). Cell clustering was conducted using the Uniform Manifold Approximation and Projection (UMAP). Differentially expressed genes were identified from normalized data using the Wilcoxon rank sum test for statistical significance. Enriched gene sets were identified using gene set enrichment analysis (GSEA). The scRNA-seq data have been deposited in the Gene Expression Omnibus (GEO) with accession number GSE295632.

### Multiplex and QPCR analysis

For multiplex cytokine assays, CD3^+^ T cells from the cerebrum of aged mice sorted by fluorescence activated cell sorting. Cells were cultured in 96-well plates pre-coated with 10 µg/ml of anti-CD3 and anti-CD28. Cell-free culture supernatant were collected after culturing for 72 hours. Concentrations of cytokines were measured using LegendPlex kits, a kit for bead-based multiplex assays (Biolegend) (29), following the manufacturer’s instructions.

For QPCR analysis, cells in the hippocampus of aged mice with treatment with anti-CD153 or isotype control were sorted by fluorescence activated cell sorting. TaqMan RT-qPCR was then performed using the one-step Cells-to-CT kit (Thermo Fisher Scientific), following the manufacturer’s instructions. This method enables direct lysis of sorted cells and reverse transcription of RNA without the need for prior RNA purification, ensuring rapid and efficient gene expression analysis.

### Statistical analysis

For scRNA-seq data, differential gene expression was assessed using the Wilcoxon rank-sum test, with a false discovery rate (FDR) < 0.05 considered statistically significant. For GSEA, results were considered significant with normalized enrichment score (NES) > 1, and FDR < 0.25. For other experiments, statistical significance between two groups was determined using either the Student’s *t*-test or one-way ANOVA followed by Tukey’s *post-hoc* test, with a threshold of *p* < 0.05.

## Results

### Aging alters the transcriptomes of CD4 and CD8 T cells in brain-associated tissue

**Figure 1:**
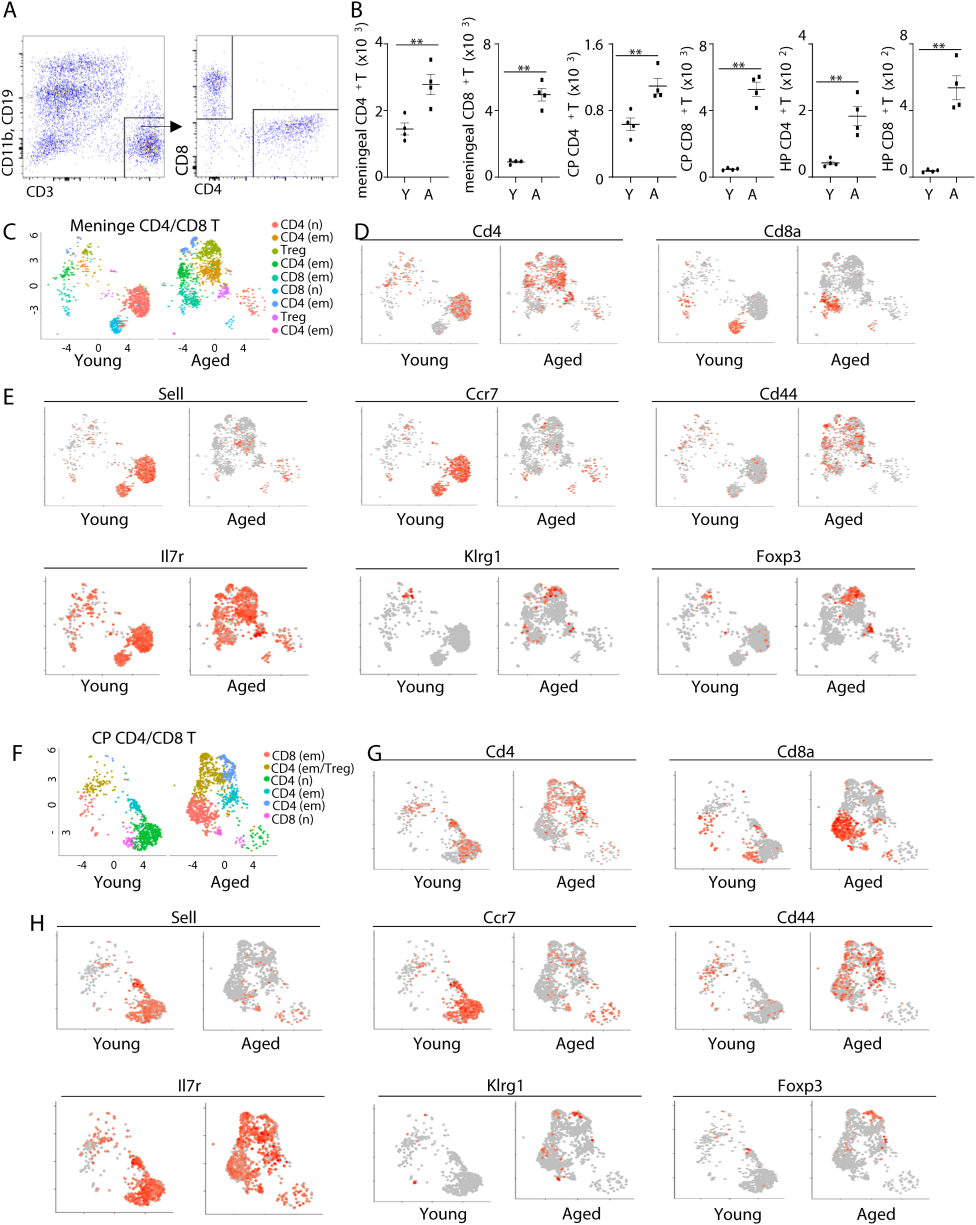
Aging alters the transcriptomes of CD4 and CD8 T cells in the meninges and the choroid plexus. **(A)** Representative flow cytometry profiles showing gating strategies of CD4 and CD8 T cells in brain-associated tissue. **(B)** Numbers of CD4 and CD8 T cells in the meninges, choroid plexus (22), and hippocampus (HP) of young (10 weeks old) and aged (18 month old) mice. **(C–H)** CD4 and CD8 T cells from the meninges and CP of young and aged mice were sorted by fluorescence activated cell sorting (FACS), and scRNA-seq were performed. **(C)** UMAP plot showing cell clustering in meningeal CD4 and CD8 T cells. **(D)** Feature plots show Cd4 and Cd8a expression in meningeal CD4/CD8 T cells. **(E)** Feature plots show expression of the indicated genes in meningeal CD4/CD8 T cells by scRNA-seq. **(F)** UMAP plot showing cell clustering in CP CD4 and CD8 T cells. **(G)** Feature plots show Cd4 and Cd8a expression in CP CD4/CD8 T cells. **(H)** Feature plots show expression of the indicated genes in CP CD4/CD8 T cells by scRNA-seq. Error bars = mean ± SEM. **p<0.01. Data are from 4 mice per group **(A, B)** and represent 2 independent experiments, or are pooled from 5 mice per group **(C-H)**.

To examine the effects of aging on the transcriptomes of CD4 and CD8 T cells in brain-associated T cells, we first examined the abundance of CD4 and CD8 T cells in the meninges, choroid plexus and hippocampus tissue by flow cytometry analysis. Interestingly, aged mice had increased abundance of both CD4 and CD8 T cells in the meninges, choroid plexus and hippocampus tissue (**Figures 1A, B**). To determine the properties of these cells, we sorted CD4 and CD8 T cells by fluorescence activated cell sorting (FACS) from young and aged mice and performed scRNA-seq. Because there were too few T cells in the hippocampus to enable robust FACS sorting, we performed scRNA-seq using meninges and choroid plexus tissue. Notably, the majority of CD4 and CD8 T cells in the meninges of young mice were naïve T cells, characterized by high expression of *Sell* and *Ccr7* (**Figures 1C–E**). These naïve T cells were diminished in aged mice (**Figures 1C-–E**). In contrast, both CD4 and CD8 T cells in aged mice exhibited an effector memory -like phenotype. They expressed high levels of *Cd44*, *Il7r* and low levels of *Klrg1* (**Figures 1C–E**). In addition, Foxp3+ Tregs were barely detected in the meninges of young mice, but they accumulated with aging (**Figures 1C–E**). UMAP analysis also indicated that the difference between cell states (naïve vs effector memory) overwhelms the difference between cell types (CD4 T vs CD8 T) (**Figures 1C–E**). Thus, aging is associated with increased effector memory T cells, but reduced naïve T cells in the meninges.

We observed similar results with scRNA-seq data with choroid plexus T cells (**Figures 1F–H**). While cells with naïve T cell transcriptomes were diminished in aged mice, cells expressing high levels of effector memory molecules (Cd44 and Il7r) were increased in the choroid plexus of aged mice (**Figures 1F–H**). Tregs also accumulated in the choroid plexus in aging (**Figures 1F–H**). At the transcriptome level, the differences between cell states were larger compared to the differences in cell types (**Figures 1F–H**). Together, aging induces a shift from naïve to effector memory T cells in both meninges and choroid plexus.

### Aging alters the transcriptomes of CD8 T cells in brain-associated tissue

**Figure 2:**
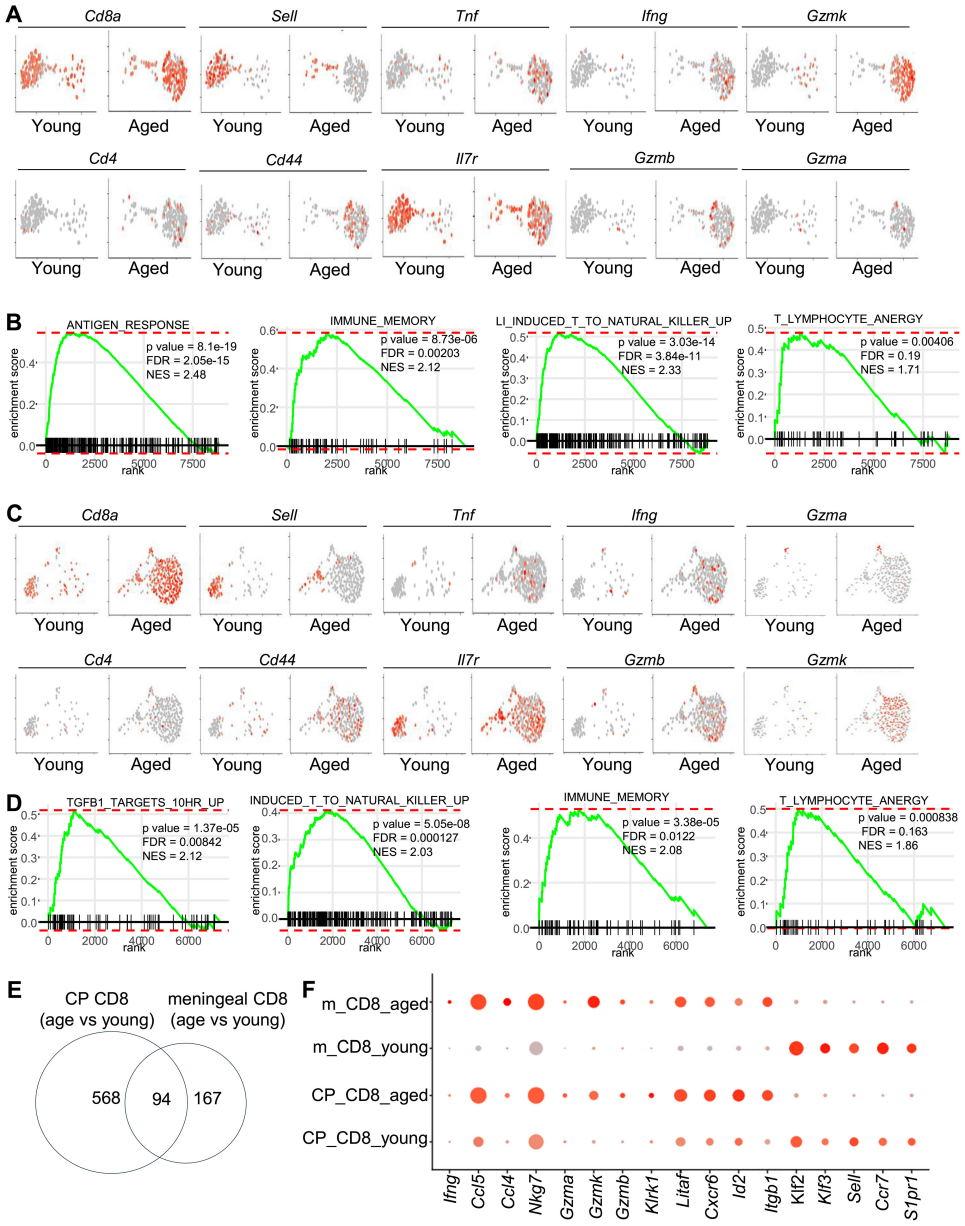
Comparison of the transcriptomes of CD8 T cells in the meninges and the choroid plexus **(A)** The transcriptomes of meningeal CD8 T cells were sorted in silico. Feature plots show expression of the indicated genes by sorted meningeal CD8T cells. **(B)** Enrichment of the indicated gene sets for genes that were expressed higher in meningeal CD8 T cells from aged mice compared to those in young mice. **(C)** The transcriptomes of choroid plexus (22) CD8 T cells were sorted in silico. Feature plots show expression of the indicated genes by sorted CP CD8T cells. **(D)** Enrichment of the indicated gene sets for genes that were expressed higher in CP CD8 T cells from aged mice compared to those in young mice. **(E)** Numbers of genes that were expressed higher with aging in meningeal CD8 T cells, and CP CD8 T cells, or both. **(F)** Dot plots show expression of the indicated genes in meningeal or CP CD8 T cells in young or aged mice. Data are pooled from 5 mice per group **(C-E)**.

We next sorted CD8 T in silico and compared the transcriptomes of CD8 T cells in young vs aged meninges and choroid plexus. In the meninges of aged mice, *Sell^+^Cd44^-^* naïve CD8+ T cells were depleted, whereas *Sell^-^Cd44^+^* CD8^+^ T cells accumulated (**Figure 2A**). These aging-associated CD8^+^ T cells expressed high levels of genes in response to antigen-responses and killer cell activity, including cytotoxic molecules Gzmb, Gzmk and cytokines Ifng and Tnf. Genes upregulated with aging in meningeal CD8^+^ T cells were also enriched in genes related with immune memory and lymphocyte anergy (**Figure 2B**). Similar results were observed in CD8+ T cells in the choroid plexus (**Figures 2C, D**). CD8+ T cells in the choroid plexus of aged mice expressed increased levels of genes related to antigen responses, cytotoxic activities, immune memory and lymphocyte anergy (**Figures 2C, D**). Indeed, around 94 genes were significantly upregulated with aging in both meningeal and CP CD8^+^ T cells (**Figure 2E**). These genes included cytotoxic molecules (Gzma, Gzmk, Nkg7), proinflammatory chemokines (*Ccl4, Ccl5*), stimulatory receptors (Klrk1), and gene markers for tissue-residency/infiltration (*Cxcr6, Itgb1*) (**Figure 2F**). These results together suggest that aging induced the accumulation of effector-memory-like tissue-resident/infiltrating CD8+ T cells in both the meninges and the choroid plexus.

### Aging is associated with accumulation of Th2 cells, Tregs and CD153-expressing CD4+ T cells in meninges and the choroid plexus

**Figure 3:**
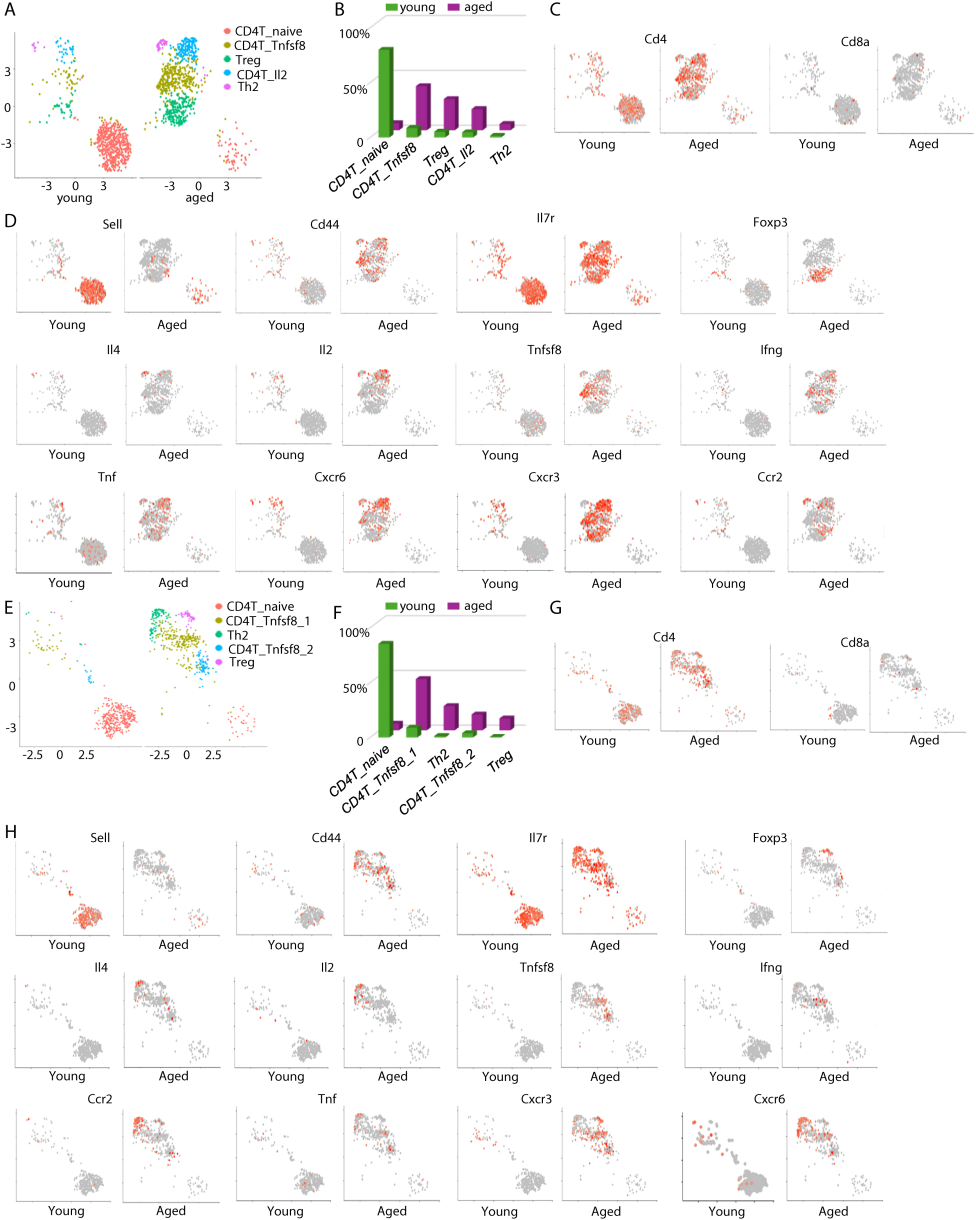
Aging induces the appearance of distinct effector CD4 T cells in the meninges and the choroid plexus. **(A)** The transcriptomes of meningeal CD4 T cells were sorted in silico. UMAP plots show cell clustering. **(B)** Frequencies of each meningeal CD4 T cell subset in young and aged mice, by scRNA-seq. **(C)** Feature plots showing expression of Cd4 and CD8a in in silico sorted meningeal CD4 T cells. **(D)** Feature plots showing expression of the indicated genes in meningeal CD4 T cells. **(E)** The transcriptomes of CP CD4 T cells were sorted in silico. UMAP plots show cell clustering. **(F)** Frequencies of each CP CD4 T cell subset in young and aged mice, by scRNA-seq. **(G)** Feature plots showing expression of Cd4 and CD8a in in silico sorted CP CD4 T cells. **(H)** Feature plots showing expression of the indicated genes in CP CD4 T cells. Data are pooled from 5 mice per group **(C-E)**.

We next sorted CD4 T cells in silico and examined their changes with aging. In the meninges, naïve CD4+ T cells were diminished with aging, while several effector T cells subsets appeared in the aged mice (**Figures 3A–D**). We annotated the aging-associated meningeal CD4+ cells based on their expression of main effector molecules (**Figure 3A**). The largest CD4+ T cell subset in the meninges of aged mice expressed the TNF superfamily gene *Tnfsf8* (**Figures 3A–D**). *Tnfsf8* encodes the protein CD153, a ligand to CD30. Tregs represented another major CD4+ T cell subset that accumulated in the meninges of aged mice (**Figures 3A–D**). In addition, Il4-expressing Th2 cells and Il2-expressing effector CD4+ T cells were also detected in the meninges of aged mice, but not those in young mice (**Figures 3A–D**). Ifng was expressed in both Il2-expressing and CD153^+^ CD4^+^ T cell subsets (**Figure 3D**). These aging-associated CD4+ effector T cells expressed distinct chemokine receptor genes (**Figure 3D**). Th2 cells expressed high levels of CCR2, whereas the other CD4+ T cells expressed Cxcr3 (**Figure 3D**). Cxcr6 is expressed highly in Th2 cells and Il2-expressing cells (**Figure 3D**). Thus, aging induced the accumulation of distinct effector CD4+ T cells in the meninges.

Similar to the CD4^+^ T cells in the meninges, CD4+ T cells in the choroid plexus in aged mice were predominantly effector T cells (**Figures 3E–H**). Two subsets of CD153^+^ (Tnfsf8^+^) CD4^+^ T cells were detected in the choroid plexus of aged mice, with one subset co-expressing Ifng (**Figures 3E–H**). Tregs and Th2 also accumulated in the choroid plexus of aged mice (**Figures 3E–H**). However, separation between Th2 cells and Il2-expressing T cells was not clear in the choroid plexus (**Figures 3E–H**). As in the meninges, Th2 cells in the choroid plexus of aged mice expressed high levels of Ccr2 and Cxcr6, whereas Tregs and CD153+ CD4+ T cells expressed Cxcr3 (**Figures 3E–H**). Thus, aging-induced CD4+ T cells in the choroid plexus were similar to those in the meninges.

### CD153+ CD4+ cells infiltrate into the hippocampus of aged mice and protect cognitive function

**Figure 4:**
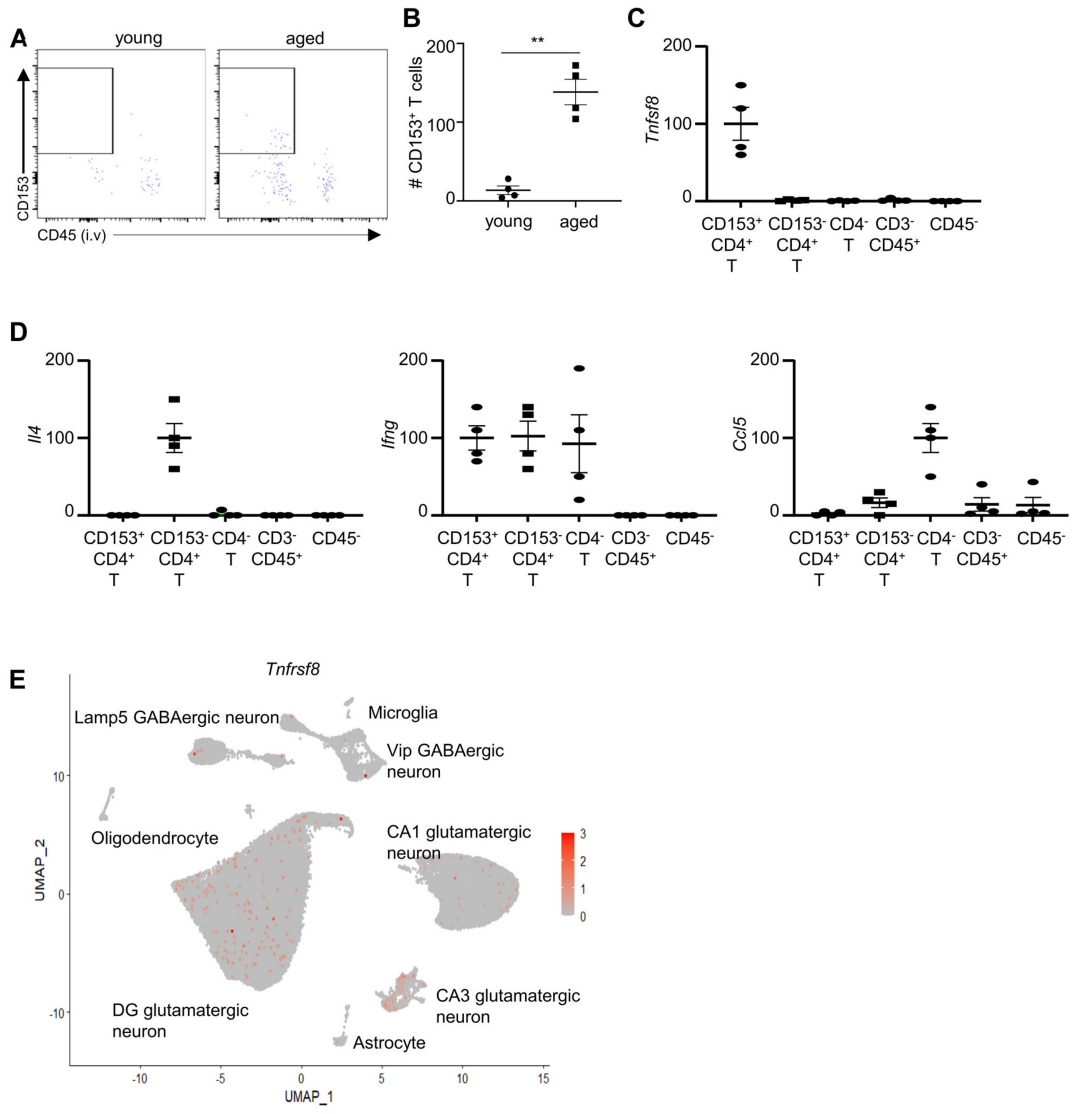
CD153+ T cells infiltrate into the hippocampus of aged mice. **(A)** Representative flow cytometry profiling showing CD153+ T cells in the hippocampus young and aged mice. Mice were administrated with anti-CD45.2-PE antibodies and euthanized after 3 minutes. Plots were re-gated on hippocampus CD4^+^ CD3^+^ CD11b^-^CD19^-^ cells as in **Figure 1A**. **(B)** Numbers of CD153^+^ CD4^+^ T cells in the hippocampus of young and aged mice. **(C)** CD153^+^CD4^+^, CD153^-^CD4^+^CD3^+^, CD4^-^CD3^+^, CD3^-^CD45^+^, and CD45^-^ cells were sorted from the hippocampus of aged mice using fluorescence activated cell sorting. Relative expression (fold change) of the *Tnfsf8* was examined by RT-qPCR analysis. **(D)** Relative expression (fold change) of the indicated genes in sorted cells in the hippocampus of aged, examined by RT-qPCR analysis. **(E)** Expression of *Tnfrsf8* across hippocampal and cortical cell populations based on a publicly available single-cell RNA-seq dataset from the Allen Mouse Brain Atlas (37). Data are from 4 mice per group, representative of two independent experiments. qPCR results were normalized to *Gapdh* **, P<0.01..

We asked whether the aging-associated CD4+ T cells might infiltrate into the brain parenchyma. We used flow cytometry analysis to examine CD4+ T cells in the hippocampus, due to its important role in cognitive function. We used an established anti-CD45.2 PE intravenous labeling assay to distinguish circulating T cells and tissue-infiltrating T cells (26). Specifically, mice were administered with anti-CD45.2 PE antibodies intravenously, and euthanized 3 mins later. Flow cytometry analysis was performed to examine the labelling of anti-CD45PE antibody in CD4+ T cells. Indeed, CD4+ cells were mostly circulating T cells in the hippocampus of young mice, indicated by positive labeling of anti-CD45 PE (**Figures 4A, B**). However, a significant proportion of CD4+ cells in the hippocampus of aged mice were not labelled by anti-CD45 PE, suggesting that they are tissue-infiltrating or resident T cells (**Figures 4A, B**). CD153^+^ CD4^+^ T cells were detected in the hippocampus of aged mice, but not in young mice (**Figures 4A, B**). These aging-associated CD153^+^ CD4^+^ T cells were tissue-infiltrating or resident T cells, demonstrated by negative labeling of anti-CD45.2PE (**Figures 4A, B**). These data suggest that CD153^+^ CD4^+^ T cells infiltrate into the hippocampus of aged mice.

Due to the limited number of hippocampus-infiltrating T cells, our attempts at single-cell RNA sequencing (scRNA-seq) were unsuccessful. Instead, we performed qPCR to assess gene expression in CD135^+^CD4^+^ T cells in the hippocampus (**Figures 4C, D**). The qPCR analysis revealed that CD135^+^CD4^+^ T cells expressed the highest levels of *Tnfsf8* in the hippocampus of aged mice, suggesting they may be the predominant source of CD153 in this region (**Figure 4C**). These cells did not express significant levels of *Il4* or *Ccl5* (**Figure 4D**). While *Ifng* was detected in CD135^+^CD4^+^ T cells, similar levels were also observed in CD135^−^CD4^+^ and CD4^-^ T cells (**Figure 4D**). Collectively, these findings indicate that CD135^+^CD4^+^ T cells are uniquely characterized by their high expression of *Tnfsf8*.

The CD153 is a TNF superfamily protein that functions by binding to CD30 (encoded by Tnfrsf8 gene) of target cells. The interaction between CD153 and CD30 requires contact dependent interaction of the cells. Indeed, analysis of a previously published dataset available from Allan Brain Map indicates that some neurons express CD30, suggesting that CD153+ cells might affect neural function (**Figure 4E**).

**Figure 5:**
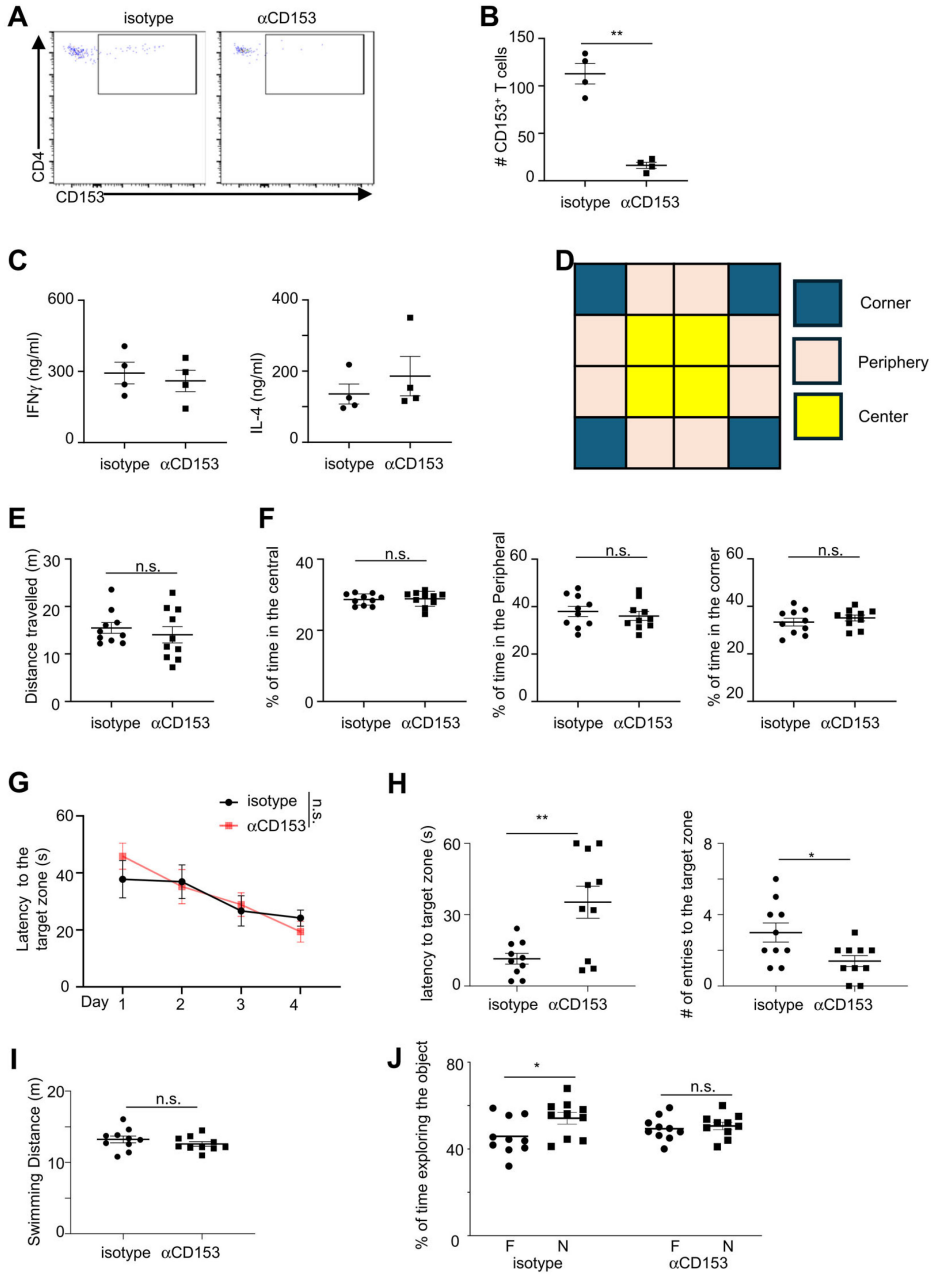
Depletion of CD153+ cells led to worse cognitive function in aged mice. **(A)** Representative flow cytometry profiling showing CD153+ T cells in the hippocampus of aged mice treated with anti-CD153 depleting antibodies (left) or isotype controls (right). **(B)** Numbers of CD153+ CD4+ T cells in the hippocampus of aged mice treated with anti-CD153 depletion antibodies or isotype controls. **(C)** CD3+ T cells were sorted from the cerebrum of aged mice treated with anti-CD153 depletion antibodies or isotype controls and cultured for 3 days with plate-bound anti-CD3 and anti-CD28 antibodies. Ifng and IL-4 concentrations were measured by LegendPlex Multiplex assays. **(D)** Virtual division of the Open Field Test arena into center, peripheral and corner zones. **(E)** Distance travelled in Open Filed Test for aged mice treated with anti-CD153 depletion antibodies or isotype controls. **(F)** Percentages of time spent in the center, peripheral, and corner zones in Open Field Test for aged mice treated with anti-CD153 depletion antibodies or isotype controls. **(G)** Escape latency in the 4-day training periods in Water Maze Test for aged mice treated with anti-CD153 depletion antibodies or isotype controls. **(H)** Latency to the target zone and entries to the target zone in the day 5 Water Maze Test for aged mice treated with anti-CD153 depletion antibodies or isotype controls. **(I)** Distance of swimming in the day 5 probe trial for aged mice treated with anti-CD153 depletion antibodies or isotype controls. **(J)** Percentages of time exploring the novel object (N) and familiar object (F) in the Novel Object Test, for aged mice treated with anti-CD153 depletion antibodies or isotype controls. Data are from 4 mice **(A-C)**, or 10 mice per group **(D-J)**, representative of 2 independent experiments. *, P<0.05; **, P<0.01; n.s., not significant.

We thus examined the role of CD153^+^ cells in regulating cognitive function using anti-CD153 antibodies. A previous report suggest that the commercial available anti-CD153 antibodies Clone RM153 may possess the capability to deplete CD153-expressing T cells (30). Using flow cytometry analyses, we found that treatment with anti-CD153 antibody RM153 depleted CD153^+^ CD4^+^ cells in aged mice (**Figures 5A, B**). Anti-CD153 antibodies did not influence Ifnγ and IL-4 production from T cells in the brain of aged mice (**Figure 5C**). Behavior tests were then performed to examine general behavior and cognitive function (**Figures 5D–J**, **Supplementary Figure 1**). Anti-CD153 antibodies did not affect the general mobility of aged mice, demonstrated by comparable distance travelled between mice treated with anti-CD153 antibodies and those treated with isotype controls (**Figures 5D, E**). The percentages of time spent in the central, peripheral and corner were also comparable between aged mice treated with anti-CD153 antibodies and those treated with isotype controls, suggesting that depletion of CD153+ cells did not affect the general stress levels of aged mice (**Figure 5F**). Aged mice treated with anti-CD153 antibodies and isotype controls performed similarly in the 4-day water maze training test, indicating that spatial learning was not affected by depletion of CD153+ cells (**Figure 5G**). However, aged mice with anti-CD153 treatment had significantly increased latencies into the target zone and reduced entries into the target zone in the day-5 probe trial, indicating reduced spatial memory (**Figure 5H**). Swimming distance was comparable between mice treated with anti-CD153 antibodies and those treated with isotype controls, indicating that CD153 treatment did not affect swim speed (**Figure 5I**). In a separate behavioral assessment using the Novel Object Test, aged mice treated with anti-CD153 antibodies failed to distinguish between the novel and familiar objects, suggesting impaired novel object recognition (**Figure 5J**). Together, these results suggest that CD153-expressing cells play a protective role in regulating cognitive function in aged mice.

## Discussion

In this study, we revealed the transcriptomic changes of CD4 and CD8 T cells with aging in the meninges and the choroid plexus. Our data show that distinct CD153^+^ subsets accumulate in both the meninges and the choroid plexus in aging. Using flow cytometry analysis, we found that these CD153^+^ cells infiltrated into the hippocampus. Experiments with depletion antibodies suggest a protective role for these cells in regulating cognitive function. Together, these results provide insights into T cell aging and suggest that some aging-associated CD4^+^ T cells may protect brain homeostasis.

In our scRNA-seq data, we found that CD153+ T cells, Tregs and Th2 cells are the main CD4+ subsets that accumulate in brain-associated tissue. Previous work indicate that Tregs also accumulated in the spleen of aged mice (8). Previous studies also reported increased CD153+ CD4^+^ T cells in the spleens and adipose tissue of aged mice, high fat diet-fed mice and mouse models with lupus (31–34). These observations suggest that the accumulation of Tregs and CD153^+^ T cells might represent a common feature of both aging and tissue inflammation. However, the accumulation of Th2 cells has not been previously observed in the spleen with aging, suggesting tissue-specific aging mechanisms for CD4+ T cells (8). Future efforts to comprehensively investigate the transcriptomic and functional similarities and differences of aging-induced T cells at various lymphoid and non-lymphoid organs might provide further insights into mechanisms of T cell aging.

CD153 exerts its function by ligation with CD30. CD30 was initially discovered to be highly expressed in B lymphoma cells (35, 36). CD30 is also found to be expressed in the spontaneous germinal center B cells of aged mice (31). CD153 has been found to be elevated in T cells in aged mice in other tissue such as the spleen (31, 32). Interaction between CD153+ CD4+ T cells with B cells in the spontaneous germinal center might promote spontaneous autoantibody production via CD153/CD30 interaction (31, 32). The CD153/CD30 signaling in the brain tissue, however, remains poorly understood. In addition, CD30 expression and CD153/CD30 signaling in non-B cell types have not been extensively explored previously. By analysis of Allan brain map datasets, we found that CD30 was expressed in hippocampus neurons. The infiltration of CD153+ T cells into the hippocampus suggests possibilities of direct interaction between CD153+ T cells and neurons via CD153/CD30 signaling. Depletion of CD153+ T cells led to worse spatial memory in aged mice, suggesting that CD153 + T cells may play a protective role in neural function. As electrophysiology studies on aged brain slices present challenges, we were unable to examine the direct effects of CD153 in regulating neuron function. In addition, due to the lack of available antibodies that can reliably detect CD30 in mouse tissue, we were not able to provide CD30 protein expression in mouse brain tissue. Future studies with new technologies and reagents to explore the precise direct effect of CD150/CD30 signaling in regulating neuron activities in aged mice would be highly worthwhile. In addition, using intravenous anti-CD45.2 PE labelling, our data show that CD153^+^ cells infiltrated into the brain parenchyma. In future investigation, it would be worthwhile to explore whether CD153^+^ T cells also infiltrate into other organs in aging organisms at homeostasis and various pathological conditions such as tumors and to explore the specific role of these unique aging-induced T cells in regulating tissue homeostasis and malignancy.

There are several additional limitations to this study. Our flow cytometric analysis indicates that treatment with the commercially available anti-CD153 clone RM153 depleted CD153^+^ T cells. Thus, this treatment may have broader effects beyond simply reducing CD153/CD30 signaling. In adipose tissue from high-fat diet-fed mice, CD153^+^ CD4^+^ T cells also expressed *Spp1* (encoding osteopontin), which may contribute to insulin resistance through osteopontin production (34). However, *Spp1* was not expressed by brain-associated CD153^+^ CD4^+^ T cells or other T cells in our scRNA-seq data. We were not able to provide feature plots for *Spp1*, because Seurat Featureplot function can only show positively expressed genes. Our scRNA-seq analysis also showed that brain-associated CD153^+^ CD4^+^ T cells expressed IFNγ, which may influence brain function. However, since other T cell subsets also produce IFNγ, and anti-CD153 treatment did not alter the overall levels of IFNγ among brain-associated T cells, it is unlikely that cytokine modulation is the primary mechanism of action. Taken together, our data suggest that CD153^+^ CD4^+^ T cells may influence brain function primarily through direct CD30/CD153 signaling rather than through changes in cytokine production. Nevertheless, we cannot rule out the possibility that CD153^+^ CD4^+^ T cells exert other functions that impact cognitive processes. Future studies employing blocking antibodies may help clarify the specific mechanisms through which these cells affect cognition. Additionally, although our qPCR data showed that CD153^+^ CD4^+^ T cells expressed the highest levels of *Tnfsf8* in the hippocampus, we cannot exclude the possibility that other cell types expressing CD153 may also influence brain function. Future efforts to address these limitations in future research would be worthwhile.

## Data Availability

The datasets presented in this study can be found in online repositories. The names of the repository/repositories and accession number(s) can be found in the article/**Supplementary Material**.
