# Supplementary material for: Aging induces T cells with distinct transcriptomic profiles and functions in brain-associated tissues
Source: Front Immunol. 2025 Jun 4;16:1619196. doi: 10.3389/fimmu.2025.1619196 (PMC12174455; doi:10.3389/fimmu.2025.1619196)
Supplement: Supplementary file 1 [file DataSheet1.pdf]

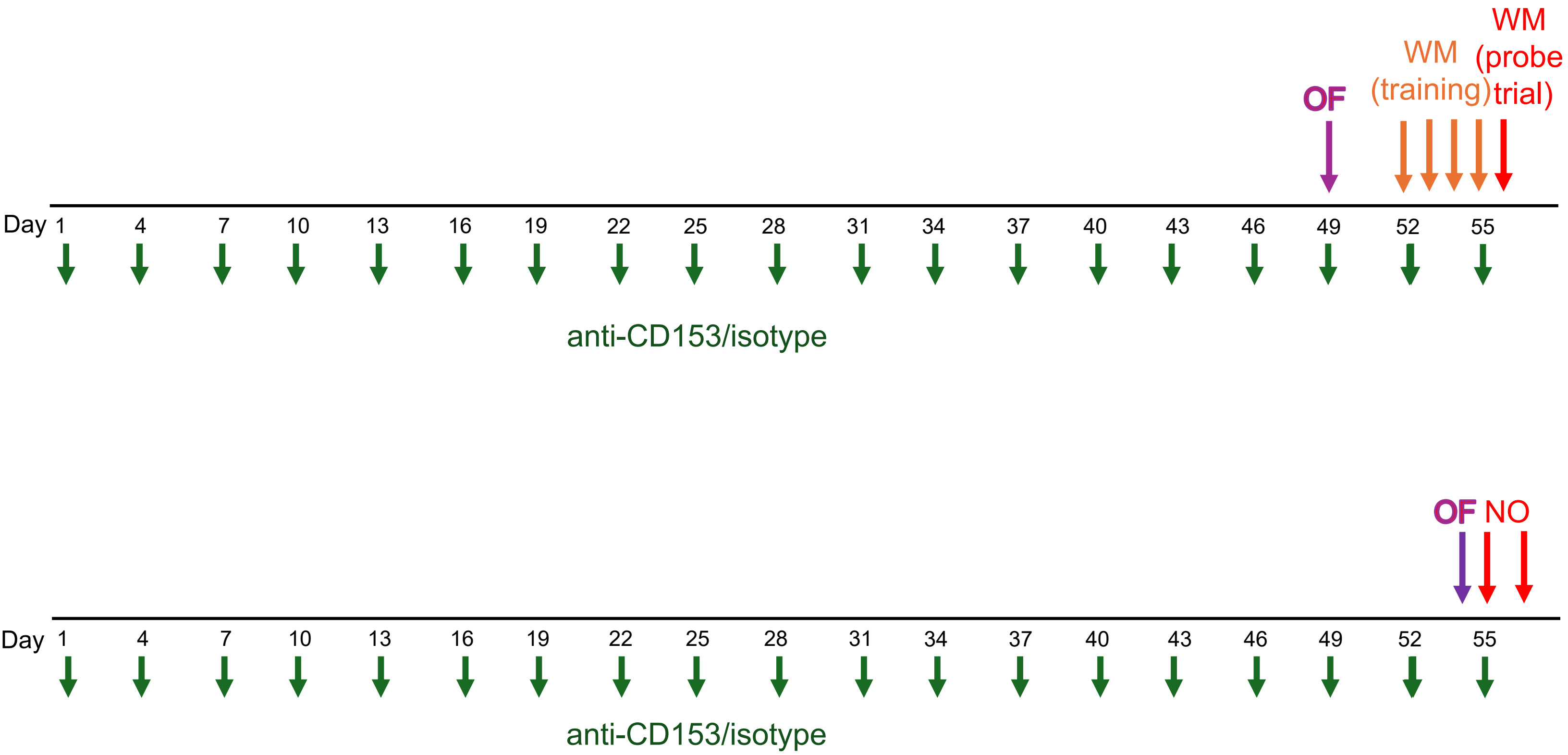

Supplementary Figure 1: **Experimental scheme for anti-CD153/isotype treatment and behavior tests.** Mice received intravenous injections of anti-CD153 antibody or an isotype control every three days for eight weeks. Mice underwent behavioral testing including open field assessment, water maze training, and probe trials, or open field and novel object recognition tasks. Behavioral testing was conducted at the conclusion of the eight-week antibody treatment period. OF: Open Field. NO: novel Object Test. WM: Water Maze Testing
